# The Possible Role of Neurofilament Light Chain as a Serum Biomarker in Anorexia Nervosa: Clinical Implications

**DOI:** 10.3390/biom15121644

**Published:** 2025-11-22

**Authors:** Andrea Amerio, Eleonora Martino, Antonella Strangio, Andrea Aguglia, Benedetta Conio, Samir Giuseppe Sukkar, Daniele Saverino

**Affiliations:** 1 Department of Neuroscience, Rehabilitation, Ophthalmology, Genetics, Maternal and Child Health (DiNOGMI), Section of Psychiatry, University of Genoa, 16132 Genoa, Italy; 2 IRCCS Ospedale Policlinico San Martino, 16132 Genoa, Italy; 3 Dietetics and Clinical Nutrition Unit, University of Genoa, 16132 Genoa, Italy; 4 Department of Experimental Medicine (DiMeS), Section of Human Anatomy, University of Genoa, 16132 Genoa, Italy

**Keywords:** anorexia nervosa, neurofilament light chain, anti-hypothalamus antibody

## Abstract

Background: Neurofilament light chain (NfL) is a well-established biomarker of neuroaxonal damage, detectable in serum through immunoassays. Its potential relevance in psychiatric conditions, including anorexia nervosa (AN), is currently under investigation. This study aims to quantify serum NfL levels in individuals with AN, evaluate their correlation with autoantibodies detection, and critically examine the specificity of NfL as a biomarker in this context. Methods: A total of 100 participants were enrolled, comprising 50 individuals diagnosed with AN and 50 age-matched, normal-weight controls. Serum concentrations of NfL and immunoglobulin G (IgG) antibodies reactive to hypothalamic antigens were measured using validated immunoassay techniques. Results: Serum NfL concentrations were markedly higher in the AN group compared to healthy controls. Interestingly, NfL levels tended to decrease with longer disease duration and with the recovery of body mass index (BMI), indicating a possible association between clinical improvement and reduced neuroaxonal damage. Furthermore, the results confirmed the presence of anti-hypothalamic autoantibodies and revealed a positive correlation between their levels and serum NfL concentrations. Conclusions: Clinical remission in AN appears to be linked to a decrease in both markers neuronal damage and hypothalamic autoimmunity. However, as elevated serum NfL is observed across a spectrum of neurological and psychiatric disorders, its specificity as a biomarker for AN should be further investigated. While NfL may reflect neuroaxonal injury in AN, its interpretation should be contextualized within a broader clinical and immunological framework.

## 1. Introduction

Anorexia nervosa (AN) is not merely a psychiatric disorder defined by caloric restriction and severe weight loss; it is a systemic condition with profound neurobiological consequences [1,2]. While historically framed as a behavioral and psychological illness, contemporary evidence challenges this reductionist view, revealing that AN induces structural and functional brain alterations that extend beyond the scope of psychological models. Neuroimaging studies consistently document reductions in brain volume and cortical thickness (CT) in acutely underweight individuals [1,3,4,5,6]. Although these changes appear largely reversible with weight restoration [5,7], the biological processes governing both neuronal injury and recovery remain elusive [8,9]. This uncertainty underscores a critical gap: the absence of reliable biomarkers capable of capturing the dynamic interplay between nutritional status, neural integrity, and systemic physiology. Addressing this gap is essential for advancing early detection, monitoring treatment response, and understanding the pathophysiological continuum of AN.

Recent research implicates immune dysregulation as a critical factor in AN pathogenesis. Elevated pro-inflammatory cytokines (IL-1β, IL-6, IL-15, TNF-α) and reduced TGF-β levels have been documented [10,11,12,13,14,15], suggesting a chronic low-grade inflammatory state. Furthermore, the presence of autoantibodies targeting hypothalamic antigens and appetite-regulating peptides introduces the possibility of immune-mediated disruption of neuroendocrine signaling, potentially reinforcing restrictive eating behaviors [14,15,16]. Meta-analytic evidence linking AN to altered bone metabolism markers (e.g., OPG, sRANKL) further supports the concept of systemic biological perturbations that may intersect with neurodegenerative pathways [13]. Collectively, these findings challenge the notion of AN as a purely psychological disorder and point toward a multifactorial model involving neuroimmune interactions.

Within this framework, blood-based biomarkers of neuronal and glial injury, such as neurofilament light chain (NfL), tau protein, and glial fibrillary acidic protein (GFAP), have emerged as promising tools for characterizing brain alterations in AN [17,18,19,20]. NfL and tau levels are consistently elevated during the acute phase and decline with BMI recovery [18,19], suggesting a reversible component of neuroaxonal damage. However, GFAP findings remain inconsistent, likely reflecting methodological heterogeneity [21,22,23]. These biomarkers, validated in neurological disorders, may provide a biological correlate to MRI-detected brain changes, offering a window into the cellular processes underlying structural alterations [24,25,26]. Yet, their application in psychiatric populations remains limited [27].

Despite its sensitivity, NfL lacks disease specificity. Elevated concentrations are observed across a spectrum of neurological and neurodegenerative conditions—including Alzheimer’s disease, multiple sclerosis, and amyotrophic lateral sclerosis (ALS)—as well as in certain psychiatric disorders [28,29]. Interpretation of NfL levels must also consider confounding factors such as age, renal function, and blood–brain barrier integrity, which influence clearance and serum concentrations [28]. Nevertheless, growing evidence linking NfL to neuroinflammatory processes, including correlations with cytokines such as IL-1β and IL-6, suggests that its elevation may reflect shared mechanisms of neuronal vulnerability across disorders [30,31]. This duality—high sensitivity but low specificity—positions NfL as a valuable, though context-dependent, biomarker that requires integration with other clinical and biological indicators.

The primary objective of this study is to investigate, within an Italian cohort of patients with AN, whether serum NfL levels are elevated and how they evolve across the course of illness and during nutritional rehabilitation. By examining the trajectory of NfL in relation to BMI restoration, we aim to determine whether this biomarker can serve as a dynamic indicator of neuroaxonal injury and recovery. Beyond this, the study explores a novel and clinically relevant question: the potential association between NfL concentrations and the presence of autoantibodies directed against hypothalamic neurons. Such an association would suggest that neuronal damage in AN is not solely a consequence of metabolic stress but may also involve immune-mediated mechanisms targeting appetite-regulating centers. This integrated perspective could redefine our understanding of AN pathophysiology, shifting it from a purely behavioral model toward a neuroimmune framework. By addressing these questions, the study seeks to provide mechanistic insights into the interplay between malnutrition, neurodegeneration, and autoimmunity, while evaluating the translational potential of NfL, alone and in combination with immunological markers, as a tool for disease monitoring, prognosis, and personalized therapeutic strategies.

## 2. Materials and Methods

### 2.1. Study Population

Between October 2019 and June 2025, 50 patients with AN were enrolled in the study. Participants received a diagnosis of AN based on the criteria specified in the Fifth Edition of the Diagnostic and Statistical Manual of Mental Disorders (DSM-5) [31]. Of the 50 participants, 42 had the restricting subtype of AN, while 8 presented with the purging phenotype (vomiting or laxative use). All participants were female, aged between 18 and 62 years, with a mean age of 20. The participants’ BMI was 15.1 ± 2.0. All patients were characterized by AN as the primary diagnosis, and all secondary diagnoses due to other medical and/or psychiatric conditions were excluded. A control group of 50 females, matched for age and gender, was also included in the study. These individuals were selected because they were not affected by eating disorders or autoimmune diseases. Their average age was 26, and their BMI was 22.3 ± 0.6. Blood samples were obtained in the morning, between 7:30 and 9:30 a.m., prior to breakfast and following a minimum fasting period of six hours. Serum was separated by centrifugation at 2000× *g* for 10 min at 4 °C and subsequently stored at −80 °C until analysis. Freezing and thawing were avoided. Finally, a follow-up of 20 patients was performed at diagnosis and 6 months later, during the recovery (BMI ≥ 18.5). The study was approved by the Ethical Committee of the Istituto di Ricovero e Cura a Carattere Scientifico (IRCCS) Ospedale Policlinico San Martino (CER 82/13 Emend. 028), and all participants provided written informed consent. The study was conducted in accordance with the Declaration of Helsinki II.

### 2.2. NfL Assay

Serum NfL concentrations were measured by an enzyme-linked immunosorbent assays (ELISA, EMELCA Bioscience Kapucinessenstraat 30; B-2000 Antwerp, Belgium), following manufacturers’ instructions, with all samples from AN and controls analyzed in the same batch to minimize inter-assay variability. This ELISA is validated for serum, plasma, and CSF. The lower limit of quantification was <6.1 pg/mL, and intra- and inter-assay coefficients of variation (CV) were <10%. All samples were measured in duplicate, and the mean value was used for analysis. Quality control samples and calibrators were included on each plate.

### 2.3. ELISA Protocol for Anti-Hypothalamus Autoantibodies Detection

Serum samples from both AN patients and healthy controls were assessed for IgG antibodies targeting hypothalamic antigens using an in-house direct ELISA protocol [32]. Briefly, 96-well plates were coated with bovine hypothalamic lysate and subsequently blocked with bovine serum albumin to minimize non-specific binding. Diluted serum samples were then applied and incubated overnight. Detection was performed using horseradish peroxidase (HRP)-conjugated anti-human IgG, followed by the addition of a chromogenic substrate. A calibration curve was generated using purified human IgG to quantify antibody concentrations. Optical density values obtained from the samples were converted into IgG concentrations (µg/mL) based on this standard curve. The intra-assay CV was 5%, while the inter-assay CV was 9.3%. Variability among triplicates remained below 10% for all reported measurements.

### 2.4. Statistical Analysis

Data normality was evaluated using the D’Agostino–Pearson test [33], which combines measures of Skewness and Kurtosis to assess how closely the dataset approximates a Gaussian distribution. A single *p*-value is generated based on the combined deviation of these parameters from the expected normal distribution.

Comparisons of autoreactive IgG and NfL marker levels between groups were performed using the Mann–Whitney U test. The Wilcoxon signed-rank test was applied to examine differences in parameter concentrations according to disease duration (less than or greater than three years). Correlations among variables in AN patients were assessed using Spearman’s rank correlation analysis. Statistical significance was set at *p* < 0.05. All analyses were conducted with GraphPad Prism version 6.0 (GraphPad Software Inc., La Jolla, CA, USA).

## 3. Results

### 3.1. Characteristics of the Study Population

The study included 50 women with anorexia nervosa (AN), aged 18–62 years, and 50 healthy female controls (CG) aged 18–51 years. Table 1 provides a summary of their characteristics, stratified by body mass index (BMI). In brief, the mean BMI was 15.1 ± 2.0 kg/m^2^ in the AN group and 22.3 ± 1.5 kg/m^2^ in the control group, with a statistically significant difference (*p* < 0.001).

### 3.2. Serum NfL Levels in AN and Controls

**Figure 1:**
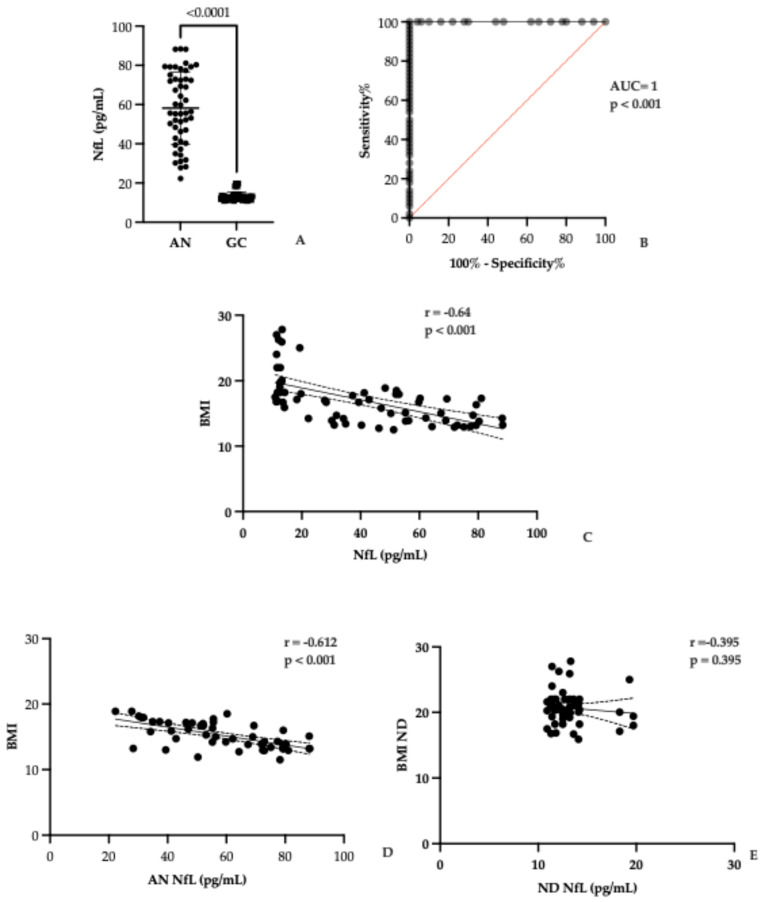
Comparison of serum NfL concentrations between individuals with anorexia nervosa (AN) and healthy controls (GC). (**A**) NfL levels (pg/mL) are plotted for each group, with individual data points shown as black dots and group means indicated by horizontal lines. A statistically significant elevation in NfL levels is observed in the AN group. (**B**) ROC curve analysis shows an excellent discriminative ability of NfL measurement between AN patients and normal donors (ND): AUC = 1 and *p* < 0.001. (**C**) Scatter plot illustrating the inverse relationship between body mass index (BMI) and NfL levels across participants. Each dot represents an individual data point. A negative correlation is evident, with a fitted regression line and 95% confidence intervals depicted as dashed lines. (**D**) Scatter plot displaying the inverse correlation between BMI and NfL levels in AN patients. (**E**) Finally, the absence of correlation between NfL levels and BMI in the group of normal donors is evident.

As shown in Table 1 and Figure 1, serum NfL concentrations were markedly higher in patients with AN compared to healthy controls, revealing a clear biological distinction between the two groups. Specifically, NfL levels in the AN cohort ranged from 22.25 to 88.37 pg/mL, with a mean of 58.18 pg/mL (±18.44 St. Dev.), whereas controls exhibited a much narrower range (10.90–19.70 pg/mL) and a substantially lower mean of 13.39 pg/mL (±2.22 St. Dev.). This difference was highly significant (*p* < 0.001) and visually evident in Figure 1A, which demonstrates minimal overlap between distributions, underscoring the robustness of this biomarker in differentiating pathological from physiological states.

In addition, no difference emerged between the restrictive and purging subtypes (*p* > 0.5). However, the latter subgroup represents only 16% of the total patients enrolled in the study.

Given the minimal overlap observed in NfL levels between AN patients and normal donors, a ROC curve analysis was performed to establish a potential cutoff point for suspecting AN in a measurement. NfL values measured in AN patients are significantly higher compared to normal donors (AUC = 1, *p* < 0.001), so the ideal cutoff is 21 pg/mL (calculated using GraphPad Prism).

Beyond group comparisons, correlation analysis revealed a strong inverse relationship between NfL levels and BMI (r = −0.64, *p* < 0.001), as illustrated in Figure 1C. This finding suggests that neuroaxonal injury in AN is not a static phenomenon but dynamically linked to nutritional status: the lower the BMI, the greater the neuronal compromise. Such a pattern reinforces the hypothesis that malnutrition exerts a direct neurotoxic effect, potentially mediated by metabolic stress, neuroinflammation, or both.

Taken together, these results position NfL as more than a passive marker of neuronal damage; its sensitivity to BMI variations indicates potential utility as a real-time indicator of disease severity and recovery trajectory. While not disease-specific, its ability to capture the neurobiological burden of AN provides a compelling rationale for its integration into clinical monitoring protocols, particularly when combined with other biomarkers and neuroimaging data.

In addition, we did not observe any correlation between the age of AN patients and serum NfL concentrations (r = 0.017, *p* = 0.905).

**Figure 2:**
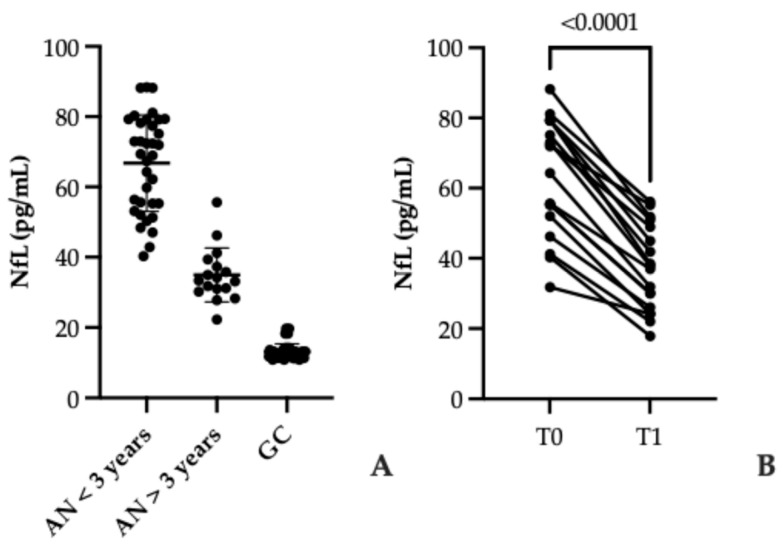
Serum NfL levels are decreasing over time to diagnosis. (**A**) Scatter plot illustrating serum NfL concentrations (pg/mL) across three groups: individuals with AN of less than 3 years since diagnosis (AN < 3 years), those with more than 3 years duration (AN > 3 years), and healthy controls (GC). Each dot represents an individual data point, with horizontal lines indicating group means. Statistically significant elevated NfL levels are observed in the AN < 3 years group compared to the other groups. (**B**) Paired plot showing longitudinal changes in NfL levels between two time points (T0 at diagnosis and T1 six months after BMI normalization) within the same individuals. Lines connect each participant’s measurements, revealing a significant reduction in NfL levels over time.

The duration of illness emerged as a critical determinant of serum NfL concentrations in patients with AN, revealing important insights into the temporal dynamics of neuroaxonal injury. Patients diagnosed within the past three years exhibited markedly higher NfL levels (range: 40.28–88.37 pg/mL; mean: 66.75 ± 13.70 St. Dev.) compared to those with a longer disease history (range: 22.25–55.61 pg/mL; mean: 34.93 ± 7.68 St. Dev.), a difference that was highly significant (*p* < 0.001). This pattern, illustrated in Figure 2A, suggests that the early phase of AN may represent a period of heightened neuronal vulnerability, possibly driven by acute metabolic stress, neuroinflammation, and hormonal dysregulation.

Importantly, this decline in NfL over time appears to parallel improvements in nutritional status, as indicated by BMI normalization (≥18.5), reinforcing the hypothesis that neuronal damage in AN is at least partially reversible. However, the persistence of elevated NfL levels in patients with longer disease duration, even after weight restoration, signals that structural or functional brain alterations may endure beyond clinical recovery. This finding challenges the assumption that nutritional rehabilitation alone fully restores neurobiological integrity and raises concerns about residual cognitive or neuroendocrine deficits.

Longitudinal follow-up data (Figure 2B) further strengthen this interpretation: NfL concentrations decreased significantly six months after diagnosis (from 61.69 pg/mL ± 18.32 St. Dev. to 36.03 pg/mL ± 12.07 St. Dev.; *p* < 0.001), demonstrating a measurable trajectory of neuronal recovery. Nevertheless, the incomplete normalization of NfL underscores the need for extended monitoring and possibly adjunctive interventions targeting neuroprotection. Collectively, these results position NfL as a dynamic biomarker that not only reflects disease severity but also captures the neurobiological impact of treatment over time, an attribute of considerable clinical relevance for prognosis and personalized care.

### 3.3. Elevated Serum Anti-Hypothalamus Autoantibodies in Anorexia Nervosa: Association with Disease Duration and Nutritional Recovery

**Figure 3:**
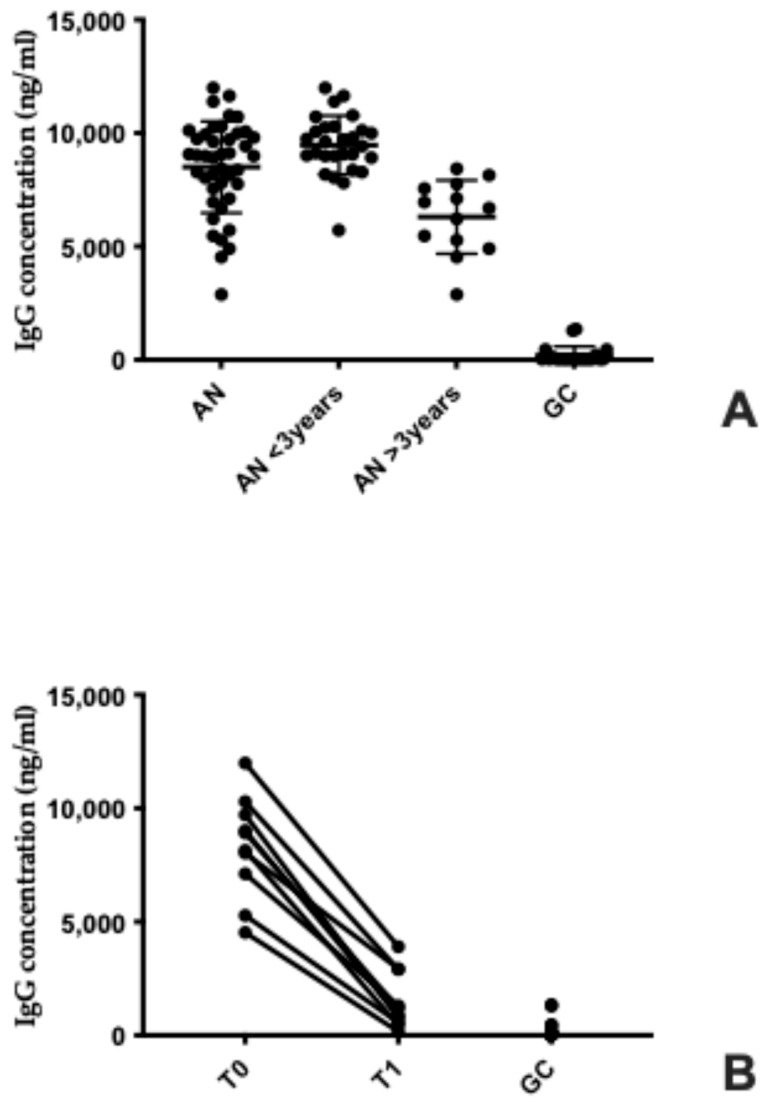
Serum anti-hypothalamus autoantibodies in AN: association with disease duration and nutritional recovery. (**A**) Scatter plot depicting serum IgG concentrations (ng/mL) across four groups: individuals with acute AN, subdivided into those with disease duration less than 3 years (AN < 3 years) and more than 3 years (AN > 3 years), and healthy controls (GC). Each dot represents an individual sample, with group means indicated. IgG levels are significantly elevated in all AN subgroups compared to the GC group. (**B**) Paired line plot showing longitudinal changes in IgG concentrations from time point T0 (at diagnosis) to T1 (six months after BMI normalization) within the same individuals. Each line connects measurements from T0 and T1 for a single participant. A general decrease in IgG levels is observed over time, while the GC group maintains consistently low concentrations.

Serum concentrations of anti-hypothalamus IgG autoantibodies were dramatically elevated in patients with AN compared to healthy controls, highlighting a robust immunological signature associated with the disorder. AN patients exhibited mean levels of 8501 ng/mL (±2023 St. Dev.; range: 2883–11,988), whereas controls showed negligible values (mean: 207 ng/mL ±392 St. Dev.; range: 30–1350), a difference that was highly significant (*p* < 0.001) (Figure 3A). This stark contrast underscores the potential role of autoimmune mechanisms in AN pathophysiology, particularly in targeting hypothalamic structures critical for appetite regulation.

Stratification by disease duration revealed an additional layer of complexity: patients with a shorter illness history (<3 years) and lower BMI (<17) displayed markedly higher autoantibody titers (9471 ng/mL ± 1294 St. Dev.) compared to those with longer disease duration and partial weight recovery (>3 years; BMI >17), whose levels, although reduced (6303 ng/mL ± 1616 St. Dev.), remained significantly above those of controls (*p* = 0.001). This gradient suggests that autoimmune activation is most pronounced during the acute phase of AN and may attenuate with nutritional rehabilitation, yet does not fully normalize, indicating a persistent immunological imprint even after clinical improvement.

Longitudinal follow-up of 20 patients further supports this interpretation: autoantibody concentrations declined substantially over six months, from 8305 ± 2243 ng/mL at diagnosis to 1510 ng/mL ± 1271 St. Dev. post-recovery (*p* = 0.002) (Figure 3B). Although these values approached those of healthy individuals, they remained significantly elevated (*p* = 0.003), suggesting incomplete immunological resolution. This persistence raises critical questions about whether residual autoimmunity contributes to relapse risk or long-term hypothalamic dysfunction. Collectively, these findings position anti-hypothalamic autoantibodies as more than an epiphenomenon, they may represent a mechanistic link between malnutrition, neuroendocrine disruption, and chronicity in AN.

### 3.4. Investigation of a Possible Correlation Between NfL and Autoantibody Levels

**Figure 4:**
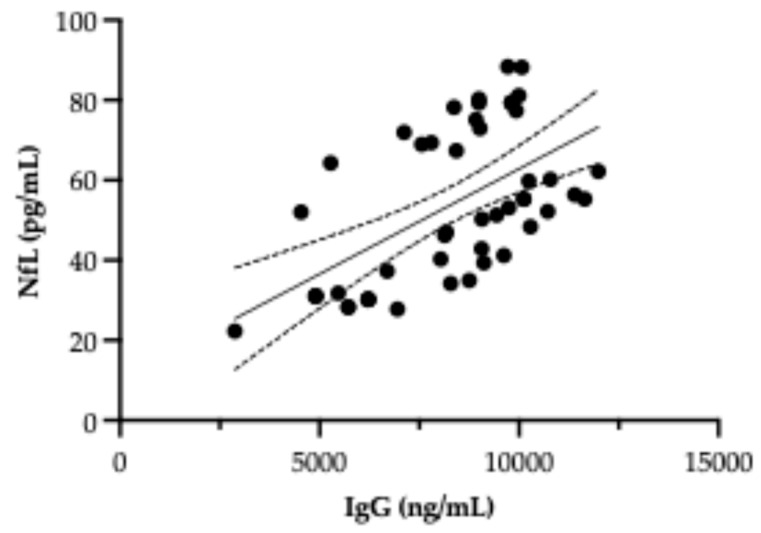
Analysis of the correlation between NfL and autoantibody levels. Scatter plot illustrating the relationship between serum IgG concentrations (ng/mL) and NfL levels (pg/mL). Each black dot represents an individual data point. The solid line denotes the best-fit linear regression, while the dashed lines indicate the 95% confidence interval. Results revealing a potential association between immune activation and neuronal injury.

Preliminary analysis of Figure 4 suggests a potential positive correlation between serum NfL levels and anti-hypothalamus autoantibody concentrations in patients with AN. Statistical analysis confirmed a significant correlation between these two biomarkers (R^2^ = 0.33, *p* < 0.001), indicating that higher autoantibody levels are associated with increased NfL concentrations. This relationship may indicate that both biomarkers, NfL and anti-hypothalamus IgG autoantibodies, could be involved in the pathophysiological processes affecting hypothalamic nuclei responsible for appetite regulation. The co-elevation of these markers in AN patients suggests a shared or interdependent mechanism contributing to hypothalamic dysfunction, potentially linking neurodegeneration and autoimmunity in the context of chronic malnutrition. However, further studies with larger sample sizes and longitudinal designs are warranted to clarify the nature and directionality of this association.

## 4. Discussion

### 4.1. NfL as a Marker of Neuronal Injury in AN

Our findings provide compelling evidence that serum NfL levels are significantly elevated in patients with AN compared to healthy controls, reinforcing previous observations [13,28,29,34,35] and strengthening the hypothesis that chronic malnutrition exerts a measurable neurobiological toll. This elevation likely reflects neuroaxonal injury, as suggested by the strong inverse correlation with BMI and the well-documented association between NfL and cortical thinning in neuroimaging studies. These data converge on a critical insight: neuronal integrity in AN is not merely compromised by psychological stressors but is profoundly influenced by metabolic deprivation and systemic physiological imbalance.

Experimental evidence from animal models further substantiates this interpretation, demonstrating that starvation induces both NfL elevation and microglial activation, hallmarks of neuroinflammatory processes. This suggests that nutritional deprivation may act as a dual insult, combining metabolic stress with immune-mediated mechanisms that accelerate structural brain changes. Such a framework challenges traditional views of AN as a purely behavioral disorder and positions it within a broader neuroimmune paradigm.

Equally important is the observation that NfL levels decline with disease duration and BMI recovery, indicating that neuroaxonal damage in AN is at least partially reversible. This dynamic trajectory aligns with neuroimaging studies showing restoration of cortical thickness following nutritional rehabilitation, underscoring the brain’s capacity for structural recovery when metabolic homeostasis is restored. However, the persistence of elevated NfL in some patients even after weight normalization raises critical questions about residual vulnerability and the potential for long-term cognitive or neuroendocrine sequelae.

Taken together, these findings highlight NfL as more than a static marker of neuronal injury; it emerges as a dynamic biomarker capable of capturing both the acute neurotoxic effects of malnutrition and the reparative processes triggered by recovery. This dual role has significant clinical implications for monitoring disease severity, guiding treatment intensity, and predicting outcomes in AN.

Finally, the group of patients analyzed is weighted toward the restrictive phenotype (84%, 42 out of 50 total patients). This may represent a limitation of the study, which we aim to address with future patient recruitment. However, based on the results obtained (16% of AN patients analyzed exhibiting purging), it can be hypothesized that there are no significant differences in serum NfL levels between the different AN subtypes.

### 4.2. Specificity and Limitations of Serum NfL

Although NfL is widely recognized as a highly sensitive biomarker of neuroaxonal injury, its clinical utility is constrained by a fundamental limitation: lack of disease specificity. Elevated serum NfL reflects a generalized process of neuronal damage rather than a disorder-specific signature, and this phenomenon has been documented across a broad spectrum of neurological and psychiatric conditions, including multiple sclerosis, Alzheimer’s disease, frontotemporal dementia, amyotrophic lateral sclerosis (ALS), traumatic brain injury, stroke [36,37,38], and status epilepticus [39]. In addition, elevated NfL was indicated as a biomarker for neurological dysfunction in chemotherapy-induced peripheral neuropathy and in myalgic encephalomyelitis-chronic fatigue patients [40,41]. Even within psychiatry, disorders such as major depressive disorder, bipolar disorder, and schizophrenia exhibit increased NfL levels, albeit typically at lower magnitudes [29,34,42]. In addition, NfL has been suggested as a reliable biomarker for the diagnosis of other neurological conditions without overt symptoms, such as minimal hepatic encephalopathy [43]. Similarly, NfL has been reported as a biomarker of depression [44]. All this suggests the potential role of NfL as an early biomarker in AN. However, this overlap highlights the need to interpret NfL within a broader diagnostic context rather than as a stand-alone marker.

Adding to this complexity, NfL concentrations are influenced by multiple physiological and clinical variables, including age, renal clearance, BMI, and comorbid conditions [29]. These confounders can significantly alter serum levels, complicating the attribution of elevated NfL solely to disease-related neurodegeneration. Furthermore, recent evidence suggests that circulating NfL may consist of degradation fragments rather than intact protein, a factor that could affect assay sensitivity and the biological interpretation of results [28]. Such nuances highlight the importance of methodological rigor and standardization in biomarker research.

Despite these limitations, the value of NfL in AN should not be underestimated. Its sensitivity to neuroaxonal injury, combined with its dynamic response to nutritional rehabilitation, positions it as a powerful adjunct marker when integrated into a multimodal framework. Rather than serving as an isolated diagnostic tool, NfL should be considered alongside complementary biomarkers, such as cytokine profiles, neuropeptides, and autoantibodies, to construct a more comprehensive picture of disease activity and recovery [43]. This integrative approach could enable biomarker-assisted stratification, improve risk prediction, and guide personalized therapeutic interventions. Ultimately, the challenge lies not in dismissing NfL for its lack of specificity, but in leveraging its strengths within a context-sensitive, multi-parameter diagnostic strategy.

Although plasma is the most used matrix for NfL quantification due to its stability and standardization across studies, recent evidence suggests that serum may serve as a reliable alternative. Several comparative studies have demonstrated a strong correlation between NfL levels in serum and plasma, despite minor differences in absolute concentrations. Recent studies [45,46] reported high concordance between serum and plasma NfL measurements using the Simoa platform, supporting the feasibility of serum-based assays in both research and clinical settings. Similarly, Bridel et al. [47] confirmed that while serum NfL levels tend to be slightly higher, they remain highly correlated with plasma values, allowing for interchangeable use in longitudinal studies. Specifically, for AN, there are currently very few studies analyzing NfL in this population, and to our knowledge, no one comparing plasma versus serum. However, a recent study measured serum NfL in AN patients [48].

Parameters investigated in this study and in our previously published, are preferentially measured in serum, which is considered the standard matrix for these biomarkers. For this reason, to harmonize our analyses, we decided to use serum for NfL quantification as well. This approach ensures consistency across all measured variables and facilitates direct comparison within AN cohort.

### 4.3. Autoantibodies and Hypothalamic Dysfunction

This study reinforces and extends previous evidence demonstrating a marked elevation of anti-hypothalamus IgG autoantibodies in individuals with AN, particularly in those with a shorter illness duration and lower BMI. This pattern suggests that autoimmune activation is not a secondary epiphenomenon but may represent an early and active contributor to disease pathophysiology. By targeting hypothalamic structures—critical hubs for appetite regulation and neuroendocrine signaling—these autoantibodies could disrupt homeostatic circuits, perpetuating restrictive eating behaviors and metabolic imbalance.

The observed positive correlation between serum NfL levels and autoantibody titers adds a crucial dimension to this interpretation. Rather than reflecting two independent processes, this association points toward a convergent pathophysiological mechanism in which immune-mediated injury and neuroaxonal degeneration are intertwined. While causality cannot be definitively established from these data, the co-elevation of these biomarkers strongly supports the hypothesis that autoimmune responses may exacerbate neuronal vulnerability, increasing hypothalamic dysfunction and potentially influencing disease chronicity and relapse risk.

These findings underscore the importance of conceptualizing AN within a neuroimmune framework rather than a purely behavioral model. They also highlight the translational potential of integrating neuroinflammatory markers such as NfL with immune profiles (e.g., cytokines, autoantibodies) to refine clinical staging and risk stratification. Such an approach could pave the way for precision medicine strategies, including biomarker-guided monitoring and the exploration of immunomodulatory interventions as adjunctive therapies in AN [13].

### 4.4. Clinical Implications

Serum NfL emerges as a promising adjunct biomarker for monitoring neuroaxonal injury in AN, particularly in severe or rapidly progressive cases where early detection of neuronal compromise is critical. Its dynamic responsiveness to nutritional status suggests potential utility in identifying patients at heightened risk for persistent neurocognitive deficits, thereby informing the intensity and timing of nutritional rehabilitation and neuroprotective strategies. Similarly, the detection of hypothalamic autoantibodies offers valuable insight into disease chronicity and immune involvement, potentially serving as a marker for patients who may benefit from immunomodulatory interventions.

However, the diagnostic value of these biomarkers is contingent upon a multidimensional assessment, since interpreting them in isolation could obscure underlying complexity and lead to erroneous conclusions. Elevated NfL and autoantibody levels must be contextualized within a comprehensive diagnostic framework that integrates clinical assessment, neuroimaging, and additional laboratory markers of neuronal injury, immune activation, and endocrine function. This multidimensional approach is essential to differentiate primary autoimmune processes from secondary effects of malnutrition, infection, trauma, or other systemic conditions. Rather than serving as stand-alone diagnostic tools, NfL and autoantibodies should be considered only as parts of a broader neurobiological profile. Relying solely on NfL as a marker for AN is a significant limitation, as it cannot capture the complex interplay between metabolic stress, immune dysregulation, and neuronal integrity. Therefore, the use of a comprehensive biomarker panel is necessary to achieve a more accurate and meaningful assessment. Such integration is pivotal for advancing precision medicine in AN, enabling clinicians to move beyond symptom-based care toward biologically informed treatment strategies.

### 4.5. Future Directions

To enhance the clinical utility of NfL and autoantibody evaluation in AN, future research should prioritize several key directions. Longitudinal studies are needed to assess the dynamics of these biomarkers throughout treatment and recovery, providing insight into their temporal patterns and prognostic value. Integrative, multimodal approaches that combine NfL with additional markers, such as tau and glial fibrillary acidic protein, alongside neuroimaging techniques may improve diagnostic specificity and help delineate underlying neuropathological processes. Mechanistic investigations into the role of autoimmunity in hypothalamic damage and appetite regulation are essential to clarify causative pathways. Furthermore, the development of reference ranges that account for age, sex, and nutritional status in psychiatric populations will be critical for accurate interpretation. Finally, identifying therapeutic targets aimed at modulating neuroinflammation and autoimmune responses may open new avenues for intervention in AN.

Combining NfL with other biomarkers (i.e., *p*-tau217, IL-6) and cognitive assessments has shown promise in improving diagnostic accuracy in neurodegenerative conditions [30]. Similar multimodal strategies could be explored in AN to enhance early detection and intervention.

Future research should leverage translational models too, such as the starvation-induced hyperactivity paradigm, to investigate mechanistic links between malnutrition, neuroinflammation, and neuronal integrity [35,48,49].

## 5. Conclusions

This study provides robust evidence that NfL levels are markedly elevated in individuals with AN, reinforcing the concept that chronic malnutrition is associated with measurable neuroaxonal injury. The observed inverse correlation between NfL concentrations and BMI, along with the significant reduction in NfL following nutritional rehabilitation, suggests that neuronal damage in AN is at least partially reversible. The observed fluctuation of NfL levels, rising during acute malnutrition and declining with nutritional rehabilitation, underscores its potential as a dynamic biomarker capable of reflecting both the extent of neuroaxonal injury and the degree of neuronal recovery. Unlike static markers, NfL provides a temporal dimension to disease monitoring, offering clinicians a tool not only for assessing current neurobiological compromise but also for tracking treatment response and predicting long-term outcomes. This adaptability makes NfL particularly valuable in a disorder like AN, where clinical improvement is often gradual and relapse risk remains high.

A distinctive contribution of this study is the demonstration of a positive correlation between NfL concentrations and anti-hypothalamic autoantibody levels, a finding that opens new interpretative pathways in understanding AN. This association suggests that neuronal injury and immune dysregulation may not be independent phenomena but interconnected processes within a shared pathogenic framework. The co-elevation of these biomarkers points toward a neuroimmune axis in which chronic malnutrition could act as a trigger for autoimmune responses targeting hypothalamic structures, thereby amplifying neuronal vulnerability. Such a mechanism provides a plausible explanation for the persistence of hypothalamic dysfunction even after partial weight restoration, which has long puzzled clinicians and researchers. By linking structural neuronal damage with immune-mediated mechanisms, this study challenges reductionist models of AN and supports a more integrated view of its chronicity and relapse risk. This perspective not only enriches the theoretical understanding of AN but also underscores the potential for novel therapeutic strategies aimed at modulating neuroimmune interactions.

As a matter of fact, from a clinical standpoint, these results highlight the potential utility of NfL as an adjunct biomarker for early detection of neurobiological compromise, risk stratification, and treatment monitoring in AN. However, it is important to emphasize that NfL is not disease-specific: its elevation is documented in numerous neurological and psychiatric conditions, including multiple sclerosis, Alzheimer’s disease, traumatic brain injury, and mood disorders. This lack of specificity limits its diagnostic value as a standalone marker. Nevertheless, its sensitivity to neuroaxonal damage makes it highly interesting to measure, particularly when interpreted alongside other clinical and biological indicators.

The integration of NfL with immunological markers, such as anti-hypothalamic autoantibody levels and/or cytokine patterns represents a novel approach that could enhance diagnostic precision and inform personalized therapeutic strategies. This combined biomarker model could help identify patients at higher risk for persistent neurocognitive deficits or treatment resistance, guiding the development of targeted interventions, including immunomodulatory therapies.

Future research should prioritize longitudinal, multimodal studies to validate these findings, establish normative reference ranges for psychiatric populations, and clarify the mechanistic pathways linking malnutrition, neuroinflammation, and hypothalamic dysfunction. Ultimately, this work underscores the importance of moving beyond purely behavioral models of AN toward an integrated neurobiological framework that accounts for both structural and immunological contributors to disease pathophysiology.

## Figures and Tables

**Table 1:** Comparison between patients with AN and healthy control group in terms of sociodemographic and serum markers.

	Anorexia Nervosa	Control Group	*p*-Value
Age (years)	20.8 ± 9.7	27.1 ± 8.61	0.45
Body Mass Index (kg/m^2^)	16.1 ± 2.0	22.3 ± 1.5	<0.001
Serum markers			
IgG autoantibody to hypothalamic cells (ng/mL)	8501 ± 2243	207 ± 392	<0.001
NfL (pg/mL)	58.18 ± 18.44	13.39 ± 2.22	<0.001

Abbreviations: IgG: immunoglobulin G; NfL: neurofilament light chain.

## Data Availability

Research data are available from the corresponding author upon reasonable request due to privacy restrictions.
